# Elective removal vs. retaining of hardware after osteosynthesis in asymptomatic patients—a scoping review

**DOI:** 10.1186/s13643-020-01488-2

**Published:** 2020-10-02

**Authors:** Barbara Prediger, Tim Mathes, Christian Probst, Dawid Pieper

**Affiliations:** 1 Institute for Research in Operative Medicine, Witten/Herdecke University, Ostmerheimer Str. 200, 51109 Cologne, Germany; 2 Hospital Gummersbach, Klinikum Oberberg GmbH, Wilhelm-Breckow-Allee 20, 51643 Gummersbach, Germany

**Keywords:** Extremity fractures, Osteosynthetic material, Internal fixations, Hardware, Remove, Retain

## Abstract

**Background:**

Osteosynthesis is the internal fixation of fractures or osteotomy by mechanical devices (also called hardware). After bone healing, there are two options: one is to remove the hardware, the other is to leave it in place. The removal of the hardware in patients without medical indication (elective) is controversially discussed. We performed a scoping review to identify evidence on the elective removal of hardware in asymptomatic patients compared to retaining of the hardware to check feasibility of performing a health technology assessment. In addition, we wanted to find out which type of evidence is available.

**Methods:**

A systematic literature search was performed in PubMed, Embase, EconLit, and CINAHL (November 2019). We included studies comparing asymptomatic patients with an internal fixation in the lower or upper extremities whose internal fixation was electively (without medical indication) removed or retained. We did not restrict inclusion to any effectiveness/safety outcome and considered any comparative study design as eligible. Study selection and data extraction was performed by two reviewers.

**Results:**

We identified 13476 titles/abstracts. Of these, we obtained 115 full-text publications which were assessed in detail against the inclusion criteria. We included 13 studies (1 RCT, 4 cohort studies, 8 before-after studies) and identified two ongoing RCTs. Nine assessed the removal of the internal fixation in the lower extremities (six of these syndesmotic screws in ankle fractures only) and two in the upper extremities. One study analysed the effectiveness of hardware removal in children in all types of extremity fractures. Outcomes reported included various scales measuring functionality, pain and clinical assessments (e.g. range of motion) and health-related quality of life.

**Conclusions:**

We identified 13 studies that evaluated the effectiveness/safety of hardware removal in the extremities. The follow up times were short, the patient groups small and the ways of measurement differed. In general, clinical heterogeneity was high. Evidence on selected topics, e.g. syndesmotic screw removal is available nevertheless not sufficient to allow a meaningful assessment of effectiveness.

## Background

Osteosynthesis is the internal fixation of fractures or osteotomy by mechanical devices (also called hardware). After bone healing, there is either the option to remove the osteosynthetic material or to leave it in place. In case the internal fixation causes symptoms (e.g. strong pain, reduced physical functioning) or complications (wound infection, device failure) the indication for removal is apparent. In contrast, the decision for or against elective removal of the osteosynthetic material in asymptomatic patients is difficult [1].

The decision for removal should be primarily based on individual patient factors (e.g. age, physical activity), considering the possible future outcomes associated with removal or non-removal [2]. The outcomes, that are important for decision-making, include chronic pain, physical functioning, complications, reoperations, negative body sensation, and spatial limitation. However, the benefits of hardware removal in asymptomatic patients are not sufficiently analysed [3, 4]. Thus, there is an ongoing debate on the justification of osteosynthetic material removal in general and on the patients groups that might benefit most by the removal [5, 6]. While some surgeons never extract the hardware in symptom free patients, other surgeons removes the hardware to prevent future complications. A national survey performed in the UK revealed that only 7% of polled surgeons had departmental or unit policies [7].

Nevertheless, removal of implants is one of the most common surgical procedures [8]. According to an analysis from Bostman et al. approximately 180 procedures per 100,000 person years could be estimated in western countries [9]. In 2018, 176,257 surgeries on hardware removal were performed in Germany, which means in about 80% of fractures treated with osteosynthesis, material was removed [10]. Similar numbers are known from the USA [11]. In Germany, it is estimated that expenses exceed about 430 Mio Euro per year [12].

Most (> 90%) of the internal fixations are removed within 24 months after the initial surgery [3]. However, there is no clear timing for removing the osteosynthetic material, the time-point of removal depends mainly on the time-point of bone healing [13]. Furthermore, the time-point of bone healing again depends on many factors including localization, type of fracture, severity, type of fixation device/s used and patient characteristic.

## Objectives

The Swiss Federal Office of Public Health delegated us to perform a scoping review to produce a basis for the decision about the feasibility of a full Health Technology Assessment (HTA). This approach is based on the idea to perform a scoping review to assess the feasibility of performing a systematic review [14]. The aim of our contracting authority (Swiss Federal Office of Public Health) was the generation of an evidence base allowing to decide about the feasibility of a full-HTA questioning if hardware removal in patients without medical indication (elective) is effective and safe compared to retaining of hardware covering all parts of the upper and lower extremities.

So far, no systematic review or meta-analysis comparing non-indicated removal of hardware to retaining of hardware in various parts of the extremities exists. However two reviews are known that specialize on syndesmotic screw removal [15, 16]. To generate an overview if there are other body parts of which a full HTA of hardware removal might be useful and to evaluate where and in what extent primary studies are needed we performed a scoping review keeping the surgical site broad. Based on the quality and quantity of the available evidence to answer the central research questions, additional or modified questions can be determined for performing a full HTA.

We performed a scoping review to identify evidence on the elective removal of hardware in asymptomatic patients compared to retaining of the hardware. In addition, we wanted to find out which type of evidence is available.

## Methods

We developed a protocol for the review (in German), which is available from the corresponding author. It was written following the structure of PROSPERO and finalized on the 30th of April 2018. We did not register the protocol anywhere. Reporting on the findings of this scoping review, we followed the PRISMA-ScR (Preferred Reporting Items for Systematic Reviews and Meta-Analyses for scoping reviews) [14]. Previously, we reported this scoping review to the Swiss Federal Office of Public Health. Considering this to be the feasibility test of performing HTA we also sighted the other domains of an HTA like costs, legal, social and organisational issues [17]. We did not find any evidence on these issues. Which is why we only concentrate on effectiveness in this manuscript. Please refer to the report provided to the Swiss Federal Office of Public Health for any detail regarding the results for other domains than effectiveness. That publication contains results from the systematic search conducted in October 2018 (containing 10 studies) only [17].

### Eligibility criteria

We included studies analysing asymptomatic patients with an internal fixation in the lower or upper extremities. All age groups were eligible. We considered the elective removal of the internal fixation device/s as experimental intervention and non-removal of the internal fixation as control intervention. All types of health outcomes were considered. This includes mortality, morbidity (pain, satisfaction, physical functioning and clinical events), clinical measures (e.g. range of motion), health-related quality of life and adverse events/complications. The inclusion was limited to studies in WHO Stratum A. This covers states with very low child and very low adult mortality including Western Europe, North-America and various Western-Pacific states [16]. We chose this criterion for two reasons. First, access to and health services are comparable in these countries as is morbidity and mortality. Transferability of technological appraisals might be restricted from countries which are non-WHO Stratum A [18]. Second is a pragmatic reason, elective removal of asymptomatic implants seems to be a novel trend in recent years in less developed countries, and we did not expect many research yet [19]. We did not define any other exclusion criteria regarding the population. We considered the following comparative study designs: randomized controlled trials, non-randomized controlled trials, cohort studies, case-control studies, before-after studies, interrupted-time-series and controlled before-after studies. We did not make any restrictions regarding publication date. We only included studies written in German or English as the reviewers could only ensure to review these languages in duplicate. We followed the framework of Arksey and O’Malley for scoping reviews [20].

### Information sources

We performed a systematic literature search in PubMed, Embase, EconLit and CINAHL (all from inception) in October 2018 and updated in November 2019. The search strategy for PubMed is displayed in Table 1. The search strategies for the other databases can be found in Additional file 1: Appendix A. The strategy was developed by our information specialist and checked by another reviewer by consulting the Peer Review of Electronic Search Strategies (PRESS) criteria [21]. We deviated from the protocol and searched the database CINAHL instead of CENTRAL due to the overlap of results within PubMed, Embase and CENTRAL.

**Table 1:** Search strategy for PubMed

osteosynthesis[tiab] OR osteosyntheses[tiab] OR osteosynthetic[tiab] OR orthopedic[tiab] OR orthopaedic[tiab] OR osteotomy[tiab] OR osteotomies[tiab] OR "Fractures, Bone"[Mesh] OR fracture[tiab] OR fractures[tiab] AND ("Fracture Fixation, Intramedullary"[Mesh] OR "Fracture Fixation, Internal"[Mesh] OR "Fracture Fixation"[Mesh] OR "Surgical Fixation Devices"[Mesh] OR "Orthopedic Fixation Devices"[Mesh] OR "Internal Fixators"[Mesh] OR "Bone Nails"[Mesh] OR "Bone Plates"[Mesh] OR "Bone Screws"[Mesh] OR "Bone Wires"[Mesh] OR material[tiab] OR materials[tiab] OR implant[tiab] OR implants[tiab] OR implantation[tiab] OR implantations[tiab] OR internal fixator*[tiab] OR intramedullary nail*[tiab] OR intramedullary fixation[tiab] OR internal fixation[tiab] OR hardware[tiab] OR plate[tiab] OR plates[tiab] OR nail[tiab] OR nails[tiab] OR screw[tiab] OR screws[tiab] OR wire[tiab] OR wires[tiab] OR pin[tiab] OR pins[tiab]) AND ("Device Removal"[Mesh] OR remov*[tiab]) NOT ("Comment" [Publication Type] OR "Letter" [Publication Type] OR "Editorial" [Publication Type]) NOT (animals[mh] NOT humans[mh])	

We performed the literature search without limiting the publication date. We applied search limitations to English and German articles and excluded comments, editorials, letters and research on animals in our electronic database search. We cross-checked all references of included articles and systematic reviews on similar topics known to us.

### Selection of sources of evidence

Records identified through the searches were added to an Endnote X7 database and duplicates were removed. All titles/abstracts identified in the electronic databases were screened by one reviewer and a second reviewer screened all excluded titles/abstracts (liberal acceleration). All articles judged to be potentially relevant were obtained. The full-texts off all potentially relevant articles were screened by two reviewers independently. Any disagreement in the study selection process was resolved in a discussion until consensus.

### Data charting process and data items

Data was collected in an a priori-piloted abstraction table by one reviewer, and the other reviewer monitored all entries for completeness and accuracy.

We extracted following study characteristics: author, publication year, region, setting, study design, recruitment period, inclusion/exclusion criteria, patient characteristics (age, body-mass index, comorbidities, fracture characteristics, surgery characteristics), time points measured and outcomes.

### Critical appraisal of individual sources of evidence

As this is a scoping review, there was no risk of bias assessment. This is consistent with guidance on the conduct of scoping reviews [20].

### Synthesis of results

We used Arksey and O’Malley’s methods and provide a descriptive analysis of the extent, nature, and distribution of the studies included in the review as well as a narrative, thematic summary of the data collected [20]. For this, we summarized the literature considering study types, location/type of the material and outcomes. On this basis, we analyzed similarities and differences within and in between studies to identify patterns and themes and postulate explanations for findings. Furthermore, we developed an evidence map illustrating the type of evidence, indications and outcomes.

## Results

### Selection of sources of evidence

**Fig. 1:**
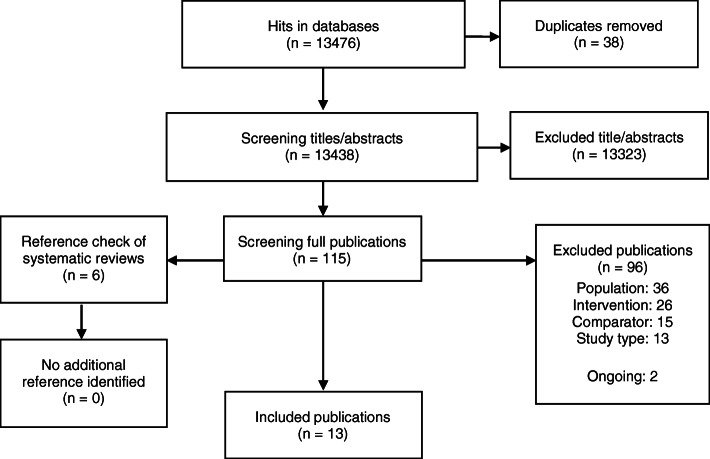
Study selection flow diagram for the literature search on all indications

Figure 1 shows the study selection process. The literature search identified 13 eligible studies [22–35]. The studies were conducted in Germany [26–28], the USA [29, 30, 32, 33], Switzerland [22, 34], Singapore [23], New Zealand [24], the UK [31] and Japan [35]. Moreover, we identified a protocol on an ongoing RCT regarding syndesmotic screw removal conducted in the Netherlands [25] and an entry on clinicaltrials.gov about an RCT regarding removal/retaining material in Lisfranc fractures conducted in Canada. There was one RCT comparing removal after three months postoperatively to non-removal [24]. We identified four cohort studies, one comparing patients with removal before weight bearing versus non removal [23] and three other comparing patients with removal with non-removal after 3 to 7 months [26, 29, 31]. Furthermore, we identified eight before-after studies, comparing the same patients before implant removal and after implant removal [22, 27, 28, 30, 32–35]. Implant removal proceeded after 3 until 27 months postoperatively. Ten studies assessed the effectiveness and safety of removal/non-removal of the osteosynthetic material at the lower extremities with six on ankle [23, 24, 29–31, 33], three on tibia [26, 27, 35] and one on femoral fractures [28]. Two studies investigated the effectiveness and safety of removal/non-removal in the proximal humerus [22, 34]. One study analysed the effectiveness of osteosynthetic material removal in children in all body parts [32].

### Characteristics of sources of evidence

The studies included adults (12 studies) [22–24, 26–31, 33–35] and children (one study) [32]. All studies compared elective removal versus non-removal of osteosynthetic material. The implants involved plates, screws, nails and staples. Sometimes a combination of implants was used. Implants were removed between 6 weeks and 27 months after surgery. Ankle screws were typically removed before weight bearing (6–12 weeks) whereas plates and nails were removed after longer periods of bone healing. The studies included a total of 588 patients. Primary efficacy and safety outcomes included functional mobility scores (e.g. Olerud-Molander ankle (OMAS) or American Orthopaedic Foot and Ankle Society ankle-hind foot scores (AOFAS)) and pain scores (e.g. visual analogue scale (VAS)). Secondary outcomes included surgery related complications, quality of life and return to work. Follow-up ranged from 9 weeks to 31 months. Table B1 in the Additional file 1 shows the detailed characteristics of the included studies.

### Results of individual sources of evidence

We identified five studies analysing pain, three of them used VAS [24, 26, 29] and the other reported pain as a count [23, 32]. Pain was measured similar in the retention and the removal groups of the cohort studies as in the before removal/after removal study from Chu et al. [32]. Except for Briceno et al. [33], all studies measured general functioning, some in various ways, functioning was assessed with a (specific) score, for example the OMAS for studies analysing syndesmotic screws, except in both studies from Gosling et al. [27, 28]. The cohort studies reported similar results in both groups for each score. According to the scores used, the before after studies showed better functional outcomes, after removal of the implant. Clinical outcomes, like range of motion, were reported in six studies [23, 24, 30, 33–35]. For the cohort studies and one before-after study, there was no difference in the outcomes [23, 24, 35] while the other three before-after studies showed advanced range of motion [30, 33, 34]. Garner et al. measured two components of the Short Form 36 scale for quality of life, with better outcomes for physical components in the removal group [26]. Please see Table 2 for individual study results.

**Table 2:** Individual study outcomes

		Follow-up (IG/CG)
**Pain**		
Bell et al. 2006 [23]	Pain free walking (*n*(%); *p*): 11(48)/2(29); *p* > 0.05	*15/16 months postoperative*^*b*^
Boyle et al. 2014 [24]	VAS (mean; MD; [95% CI]): 0.66/1.03; − 0.38; [− 1.01–0.26]	*12 months postoperative*^*b*^
Chu et al. 2009 [32]	Pain (*n*(%); *p*): 7(28)/3(12); *p* = 0.92	*16.5 months postoperative*^*b*^ *(mean)*
Garner et al. 2015 [26]	VAS (median; *p*): 0.6/0.5; *p* = 0.64	*15.4/40.6 months (median)*
Hamid et al. 2009 [29]	VAS (mean; *p*): 0.074/2.02; *p* = 0.268	*30 months postoperative*^*b*^ *(mean)*
**Functional**		
Acklin et al. 2016 [22]	Constant-Murley score (mean; MD; [95% CI]): 70.8/75.6; 4.8; [1.8–7.8].	*9 ± 4 weeks*
Bell et al. 2006 [23]	Baird and Jackson ankle score (mean, *p*): 88/86, *p* = 0.79. Return to work (*n*(%), *p*): 13(57)/4(57), *p* > 0.05	*15/16 months postoperative*^*b*^
Boyle et al. 2014 [24]	Olerud–Molander ankle score (mean; MD; [95% CI]): 86.7/82.4; 4.3; [− 5.2–13.9] American Orthopaedic Foot and Ankle Society ankle-hind foot score (mean; MD; [95% CI]): 90.1/88.6; 1.5; [− 6.0–9.1] American Academy of Orthopaedic Surgeons foot and ankle score (mean; MD; [95% CI]): 91.8/87.0; 4.8; [− 3.5–13.2]	*12 months postoperative*^*b*^
Chu et al. 2009 [32]	Pediatric Outcomes Data Collection Instrument***:*** global functioning improved *p* = 0.012	*16.5 months postoperative*^*b*^ *(mean)*
Dimitriou et al. 2020 [34]	Subjective increase of function *n*(%): 54 (96)	*12 months postoperative*^*b*^
Garner et al. 2015 [26]	Knee Outcome Survey (median; *p*): 85/78.8; *p* = 0.12 Lower Extremity Functional Scale (median; *p*): 80/66.3; *p* < 0.05	*15.4/40.6 months (median)*
Goshima et al. 2019 [35]	Japanese Orthopedic Association score (mean, SD, *p*): 93.9(7.2)/94.7(6.2); *p* > 0.05 Oxford Knee Score (mean, SD, *p*): 41.0(5.1)/43.1(4.7); *p* = 0.03	*12 months postoperative*^*b*^
Gosling et al. 2004 [28]	Complaints after nail removal (*n*(%)): 3(17)	*6.3 years (mean)*
Gosling 2005 [27]	Complaints after nail removal (*n*(%)): 10(20)	*7.4 years (mean)*
Hamid et al. 2009 [29]	American Orthopaedic Foot and Ankle Society ankle-hind foot score (mean; *p*): 85.8/85.59; *p* = 0.714	*30 months postoperative*^*b*^ *(mean)*
Miller et al. 2010 [30]	Olerud-Molander Ankle Score (mean; *p*): 42/75; *p* = 0.002 *Foot and Ankle Outcome Score (mean):* Symptoms: 58/75 Pain: 65/79 Activities of daily living: 74/87 Sports and recreation: 49/62 Quality of life: 40/53	*7 months postoperative*^*b*^
Tucker et al. 2013 [31]	Olerud-Molander Ankle Score (mean; *p*): 75.0/81.5; *p* = 0.107 Excellent overall functional outcome (%, mean adjusted difference, [95% CI]): 23.26/25; – 9.3; [− 18.5 to − 0.2]	*31 months (mean)*
**Clinical measures**		
Bell et al. 2006 [23]	*Ankle range of motion (mean motion deficit in degrees compared to normal ankle, p)* Flexion: 11.5/12.1, *p* > 0.05 Inversion: 10.4/10.0, *p* > 0.05	*15/16 months postoperative*^*b*^
Boyle et al. 2014 [24]	Ankle dorsiflexion [degree] (mean; MD; [95% CI]): 13.0/10.2; 2.7; [− 1.4–6.9] Ankle plantar flexion [degree] (mean; MD; [95% CI]): 31.2/33.6; − 2.3; [− 9.3–4.6] Calf girth loss [cm] (mean; MD; [95% CI]): 0.04/0.07M -0.21; [− 0.69–0.26] Tibiofibular clear space [mm] (mean; MD; [95% CI]):5.3/5.0; 0.34; [− 0.28–0.95]	*12 months postoperative*^*b*^
Briceno et al. 2019 [33]	Ankle dorsiflexion [degree] (mean; SE; *p*): 13.8(1.5)/10.1(2.4); *p* = 0.129. Subjective improvement of dorsiflexion (*n*(%)): 10 (48)	*3 months postoperative*^*b*^
Dimitriou et al. 2020 [34]	*Range of motion [degree] (mean; SD; p):* External rotation: 38(NR)/41(8.3); *p* = 0.07 Abduction: 125(29)/140(25); *p* = 0.001 Flexion: 130(27)/150(20); *p* = 0.001	*12 months postoperative*^*b*^
Goshima et al. 2019 [35]	*Radiological evaluation [degree] (mean; SD; p)* Hip–knee–ankle angle: 4.1(2.5)/3.9(2.7); *p* = ns Medial proximal tibial angle: 94.0(3.0)/93.7(3.0); *p* = ns Posterior tibial slope: 9.2(3.2)/9.4(3.3); *p* = ns Weight-bearing line ratio [%] (mean; SD; *p*): 67.8(10.0)/65.7(10.6); *p* = ns	*12 months postoperative*^*b*^
Miller et al. 2010 [30]	*Average range of motion [degree] (mean; p):* Dorsiflexion: 10/20; *p* < 0.05 Plantarflexion: 35/45; *p* < 0.05	*7 months postoperative*^*b*^
**Quality of life**		
Garner et al. 2015 [26]	*Short Form-36 Survey (median; p):* Mental Component Summary: 57.6/55.6; *p* = 0.78 Physical Component: 50.9/44.9; *p* < 0.05	*15.4/40.6 months median)*
**Adverse events**		
Bell et al. 2006 [23]	Syndesmotic screw malposition (*n*): 1/0 Syndesmotic screw breakage (*n*; *p*): 0^a^/2; *p* < 0.025	*15/16 months postoperative*^*b*^
Dimitriou et al. 2020 [34]	Avascular necrosis *n*(%): 7(13) Other complications *n*(%): 0(0)	*29 months postoperative* ^*b*^
Hamid et al. 2009 [29]	Syndesmotic screw breakage (*n*): 0^a^/10	*30 months postoperative*^*b*^ *(mean)*

*CG* control group, *IG* intervention group, *MD* mean difference, *SE* standard error, *VAS* visual analogue scale

^a^Screws have been removed before assessment

^b^After index procedure

### Synthesis of results

**Fig. 2:**
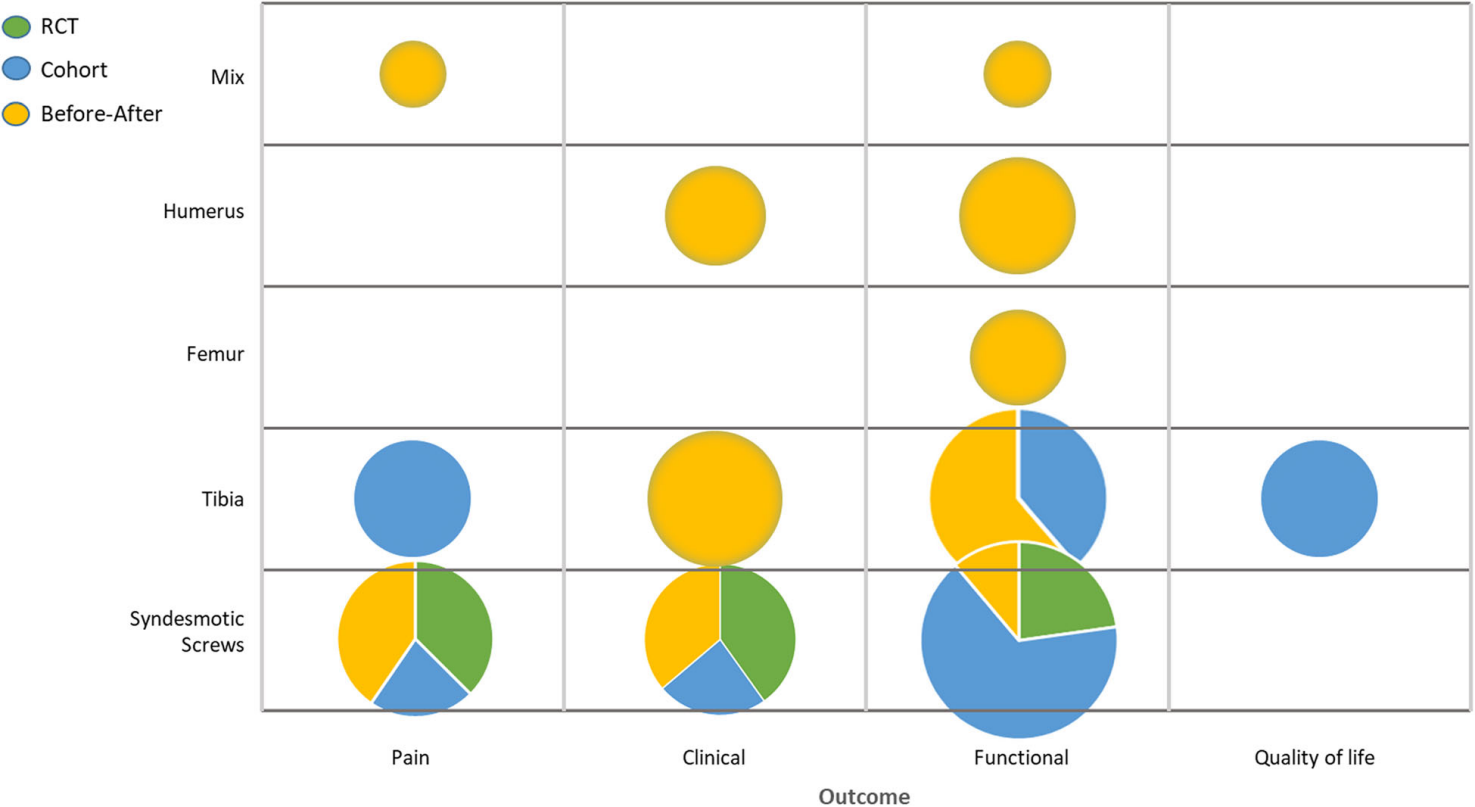
Evidence map of identified literature

Figure 2 shows an evidence map of the included literature. Six studies analysed removal/retain of syndesmotic screws. Three of them used the OMAS [24, 30, 31] and two used the AOFAS for functional assessment [24]. Three studies analysed functional outcomes of hardware removal in the tibia [26, 27, 35]. The results in general are in some parts homogenous in between different studies for the retention or removal group (e.g. functional outcomes). The other locations and types of materials differed too much to synthesise them in any way. They were all pictured as single studies.

## Discussion

### Summary of evidence

We identified 13 articles that evaluated the effectiveness/safety of hardware removal in the extremities. One of the included studies is an RCT which is about syndesmotic screw fixation in ankle fractures. There were four cohort studies examining hardware removal in the lower extremities and further eight studies with a before after design, two examining the upper extremities and one including diverse indications, the remaining examining the lower extremities. We also identified two ongoing RCT on syndesmotic screw removal and removal/retaining of material in Lisfranc fractures [25]. Three studies were conducted in Germany [26–28] and four in the USA [29, 30, 32, 35]. The other studies were conducted in Switzerland [22, 34], Singapore [23], New Zealand [24], the UK [31] and Japan [35]. The ongoing RCTs will be performed in the Netherlands and Canada [25]. There are six studies examining the removal of syndesmotic screws [23–25, 29–31, 33], two studies on plate removal from the proximal humerus [22, 34], two studies on plate removal in the tibia [26, 35], and one study on nail removal in the tibia [27] and nail removal in the femur [28]. Chu et al. included diverse indications treated with different materials. This is the only study treating children [32]. As we did not specify the outcome in this scoping review, we identified multiple outcomes concerning function, pain, clinical measures and undesired events. Three studies collected their outcomes with the OMAS as a score evaluating functional impairment, three studies used the VAS for the assessment of pain and two used the AOFAS for the functional evaluation. Moreover, there were other scores used for functional and pain evaluation. Five studies assessed flexion as a clinical outcome [23, 24, 30, 33]. There were small positive effects in the removal group for various outcomes. However, the effects differ and are not statistically significant. Four before after studies show a statistical significant effect in favour of removal regarding functional and/or clinical measures after removal [22, 30, 32, 34].

So far, the approach of asymptomatic removal of implants is based on either the wish of the patient or on habits of the surgeon/hospital policies. To help making more standardized decisions in the future, more research is needed especially considering the variety of patients and indications [7]. Along with diagnostic information, prognosis should guide clinical decision-making. Right now, decision for elective removal is partly based on prognostic factors, as for example patients with a younger age are recommended to have the hardware removed because the material will stay longer in the body [36, 37]. Research to identify prognostic factors (e.g. patient characteristics) for determining standardized decisions seems feasible and necessary.

The identified studies showed high heterogeneity regarding surgical site and patients but also regarding study design and methods. It seems feasible to perform a systematic review on either a more specific question (e.g. Is the standardized removal of syndesmotic screws effective considering pain?) or analysing other aspects like prognostic factors. Due to the high numbers of implant removal and associated costs there is a need for more valuable evidence. The underlying evidence uses too short follow-up times to guide decision-making reasonably. Furthermore, the heterogeneity of outcomes is a problem in the interpretation of results. The use of uniform or generic measurement instruments (e.g. measuring quality of life with a generic instrument) is necessary alongside with longer follow-up times. Analysing this in RCTs or cohort studies fulfilling methodological standards (e.g. considering confounding) is required and potentially feasible. Finally, the identified knowledge gaps and challenges show the need but also the possibility of performing more primary research on elective removal of hardware.

### Limitations

One of the main issues of this scoping review is that we included both, before-after and cohort studies. Interpretation of these types of studies is different and if a systematic review would be performed there should be various analyses for each study type. Methodological quality seems very heterogeneous even in the cohort studies. Above all, Dingemans et al. showed the feasibility of performing a RCT which should be the aim for future trials [25].

We limited publications to only high developed countries (WHO Stratum A) and also made restrictions to language. These restrictions could have excluded significant literature. But on the one hand, we assumed less literature on surgery of asymptomatic patients in countries other than WHO Stratum A dealing with more serious health issues and doubt in their general transferability. And on the other hand, we were not able to provide translations of other languages than English and German in the extent of a scoping review. Both should be fitted in a systematic review.

## Conclusions

We identified 13 studies that evaluated the effectiveness/safety of hardware removal in the extremities. The follow-up times were short, the patient groups small and the ways of measurement differed. In general, clinical heterogeneity was high. Evidence on selected topics, e.g. syndesmotic screw removal is available nevertheless not sufficient to allow a meaningful assessment of effectiveness.

## Supplementary information

**Additional file 1: Appendix A.** Search strategies. **Appendix B.** Characteristics of included studies.

## Data Availability

Not applicable.

## Abbreviations

AOFAS: American Orthopaedic Foot and Ankle Society ankle-hind foot score
HTA: Health Technology Assessment
OMAS: Olerud-Molander ankle score
PRESS: Peer Review of Electronic Search Strategies
PRISMA-ScR: Preferred Reporting Items for Systematic Reviews and Meta-Analyses for scoping reviews
RCT: Randomized controlled trial
VAS: Visual analogue scale
WHO: World Health Organization
