# Supplementary material for: Elective removal vs. retaining of hardware after osteosynthesis in asymptomatic patients—a scoping review
Source: Syst Rev. 2020 Oct 2;9:225. doi: 10.1186/s13643-020-01488-2 (PMC7532570; doi:10.1186/s13643-020-01488-2)
Supplement: Supplementary file 1 — Additional file 1: Appendix A. Search strategies. Appendix B. Characteristics of included studies. [file 13643_2020_1488_MOESM1_ESM.docx]

# Appendices

Appendix A: Search strategies

Embase search strategy

'osteosynthesis'/exp OR 'orthopedic surgery'/exp OR 'osteotomy'/exp OR 'fracture'/exp OR (osteosynthesis OR osteosyntheses OR osteosynthetic OR orthopedic OR orthopaedic OR osteotomy OR osteotomies OR fracture OR fractures):ti,ab,kw AND ('fracture fixation'/exp OR 'intramedullary nailing'/exp OR 'orthopedic fixation device'/exp OR 'orthopedic implant'/exp OR 'internal fixator'/exp OR 'plate fixation'/exp OR 'splinting'/exp OR 'volar plate fixation'/exp OR 'wire fixation'/exp OR 'bone nail'/exp OR 'bone plate'/exp OR 'bone screw'/exp OR 'bone wire'/exp OR 'bone pin'/exp OR (material OR materials OR implant OR implants OR implantation OR implantations OR 'internal fixator*' OR 'intramedullary nail*' OR 'intramedullary fixation' OR 'internal fixation' OR hardware OR plate OR plates OR nail OR nails OR screw OR screws OR wire OR wires OR pin OR pins):ti,ab,kw) AND ('implant removal'/exp OR remov*:ti) AND ((english OR german):la) AND ([embase]/lim) NOT (('comment' OR 'letter' OR 'editorial'):it) AND (embase NOT (embase AND medline))

EconLit search strategy

( TI (osteosynthesis OR osteosyntheses OR osteosynthetic OR orthopedic OR orthopaedic OR osteotomy OR osteotomies OR fracture OR fractures) OR AB (osteosynthesis OR osteosyntheses OR osteosynthetic OR orthopedic OR orthopaedic OR osteotomy OR osteotomies OR fracture OR fractures) ) AND ( TI (material OR materials OR implant OR implants OR implantation OR implantations OR “internal fixator*” OR “intramedullary nail*” OR “intramedullary fixation” OR “internal fixation” OR hardware OR plate OR plates OR nail OR nails OR screw OR screws OR wire OR wires OR pin OR pins) OR AB (material OR materials OR implant OR implants OR implantation OR implantations OR “internal fixator*” OR “intramedullary nail*” OR “intramedullary fixation” OR “internal fixation” OR hardware OR plate OR plates OR nail OR nails OR screw OR screws OR wire OR wires OR pin OR pins) ) AND ( (TI remov*) ) AND ( (LA (english OR german)) ) NOT ( (PT ("Comment" OR "Letter" OR "Editorial")) )

CINAHL search strategy

( MH "Osteotomy" OR MH "Orthopedic Surgery" OR MH "Fractures" OR TI (osteosynthesis OR osteosyntheses OR osteosynthetic OR orthopedic OR orthopaedic OR osteotomy OR osteotomies OR fracture OR fractures) OR AB (osteosynthesis OR osteosyntheses OR osteosynthetic OR orthopedic OR orthopaedic OR osteotomy OR osteotomies OR fracture OR fractures) ) AND ( (MH "Fracture Fixation" OR MH "Orthopedic Fixation Devices" OR MH "Internal Fixators" OR MH "Bone Screws" OR TI (material OR materials OR implant OR implants OR implantation OR implantations OR “internal fixator*” OR “intramedullary nail*” OR “intramedullary fixation” OR “internal fixation” OR hardware OR plate OR plates OR nail OR nails OR screw OR screws OR wire OR wires OR pin OR pins) OR AB (material OR materials OR implant OR implants OR implantation OR implantations OR “internal fixator*” OR “intramedullary nail*” OR “intramedullary fixation” OR “internal fixation” OR hardware OR plate OR plates OR nail OR nails OR screw OR screws OR wire OR wires OR pin OR pins) ) AND ( (MH "Device Removal" OR TI remov*) ) AND ( (LA (english OR german)) ) NOT ( (PT ("Comment" OR "Letter" OR "Editorial")) )

Additional: Pubmed search strategy for syndesmotic screws removal in ankle factures

"Ankle Fractures"[Mesh] OR ankle[tiab] OR malleolus[tiab] OR malleolar[tiab] AND ("Bone Screws"[Mesh] OR screw[tiab] OR screws[tiab]) AND ("Device Removal"[Mesh] OR remov*[tiab]) AND (english [la] OR german [la]) NOT ("Comment" [Publication Type] OR "Letter" [Publication Type] OR "Editorial" [Publication Type])

Additional: Embase search strategy for syndesmotic screws removal in ankle factures

'ankle fracture'/exp OR (ankle OR malleolus OR malleolar):ti,ab,kw AND ('bone screw'/exp OR (screw OR screws):ti,ab,kw) AND ('implant removal'/exp OR remov*:ti,ab,kw) AND ((english OR german):la)

AND ([embase]/lim) NOT (('comment' OR 'letter' OR 'editorial'):it) AND (embase NOT (embase AND medline))

Additional: EconLit search strategy for syndesmotic screws removal in ankle factures

( TI (ankle OR malleolus OR malleolar) OR AB (ankle OR malleolus OR malleolar) ) AND ( TI (screw OR screws) OR AB (screw OR screws) ) AND ( TI remov* OR AB remov* ) AND ( LA (english OR german) ) NOT ( PT ("Comment" OR "Letter" OR "Editorial") )

Additional: EconLit search strategy for syndesmotic screws removal in ankle factures

( MH "Ankle Fractures" OR TI (ankle OR malleolus OR malleolar) OR AB (ankle OR malleolus OR malleolar) ) AND ( MH "Bone Screws" OR TI (screw OR screws) OR AB (screw OR screws) ) AND ( MH "Device Removal" OR TI remov* OR AB remov* ) AND ( LA (english OR german) ) NOT ( PT ("Comment" OR "Letter" OR "Editorial")

Appendix B: Characteristics of included studies

Table B1 Characteristics of included studies

| **Study** | **Study type**  **Setting, time** | **Intervention** | **Comparison** | **Characteristics (IG/CG)** | **Outcomes, follow up** |
| --- | --- | --- | --- | --- | --- |
| Acklin 2016^21^ | Before-After Study  Switzerland, NR | N=20  Removal after 13 ± 5 months (mean ± SD) postoperatively | - | Male n (%):  8 (40)  Age [y] mean ± SD: 56 ± 12  Dominant arm n: 5  Mechanism of injury n (%): Ski 8 (40) Fall at home 4 (20) Pedestrian 4 (20) Miscellaneous 4 (20)  Operation room time [min] mean ± SD: Fracture fixation 86 ± 26  Implant removal 35 ± 10 | Constant-Murley score of the injured and contralateral shoulder  9 ± 4 weeks |
| Bell 2006^22^ | Cohort Study  Singapore, Ge-neral Hospital  01.2001-12.2002 | N=23  Removal of syndesmotic screws before weight bearing | N=7  Retaining of syndesmotic screws before weight bearing | Age [y] mean (range):  36 (18-67) / 32 (18-45)  Fracture pattern n (%): Unimalleolar 5 (22) / 2 (29) Bimalleolar 14 (61) / 4 (57) Trimalleolar 3 (13) / 1 (14) Maisonneuve 1 (4) / 0  Mechanism of injury n (%): Misstep 10 (43) / 3 (43) Sports 7 (30) / 2 (29) Fall from height 4 (17) / 0 Traffic accident 1 (4) / 2 (29) Hit over leg 1 (4) / 0  Complications n(%): Superficial wound infection 1 (4)/ 1 (14) Screw malposition 1 (4) / 0 Screw breakage 0 / 2 (29) | Baird and Jackson ankle score, pain free walking, return to work, ankle range of motion (mean motion deficit in degrees compared to normal ankle)  15/16 months postoperative^a^ |
| Boyle 2014^23^ | RCT  New Zealand, University of Otago  07.2011-11.2012 | N=26  Removal of syndesmotic screws before weight bearing,  Removal after 3 months postoperatively | N=25  Retaining of syndesmotic screws | Male n (%):  19 (73) / 16 (64)  Age [y] mean ± SD:  30.8 ± 12.8 / 36.2 ± 14.1  BMI [kg/m²] mean ± SD:  30.6 ± 4.6 / 31.9 ± 5.5  Smoker n (%):  9 (35) / 8 (32)  Diabetes n (%):  0 / 1 (4)  Ethnicity n (%): European 14 (54) / 11 (44) Maori 4 (15) / 2 (8) Pacific 6 (23) / 7 (28) Asian 2 (8) / 5 (20)  Mechanism of injury n (%): Collision sport 9 (35) / 6 (24) Non-collision sport 2 (8) / 4 (16) Simple fall 12 (46) / 13 (52) Other 3 (11) / 2 (8)  Time from injury to surgery [d] mean ± SD:  4.8 ± 4.7 / 4.0 ± 3.2  Surgical duration [min] mean ± SD):  86.3 ± 31.4 / 77.7 ± 23.5  Surgeon seniority n (%): Surgeon 1 (4) / 0 Fellow 1 (4) / 0 Senior registrar 14 (54) / 13 (52) Junior registrar 10 (38) / 12 (48)  Syndesmosis screw location from tibial plafond[mm] mean ± SD:  16.4 ± 8.4 / 19.3 ± 8.3 | Olerud–Molander ankle score, American Orthopaedic Foot and Ankle Society ankle-hind foot score, American Academy of Orthopaedic Surgeons foot and ankle score, pain, ankle dorsiflexion, ankle plantar flexion, calf girth loss, tibiofibular clear space  12 months postoperative^a^ |
| Briceno 2019^32^ | Before-After Study  USA, NR, 11.2016-03.2018 | N=21 Removal of syndesmotic screws after approximately 3 months postoperative | - | Male n(%):  8 (33)  Age [y] mean ± SD:  52 ± 16.7  Type of injury n(%):  Rotational ankle fracture 24(100)  Lauge-Hansen classification n(%):  SER 19 (79)  PER 4 (17)  PAB 1 (4)  Screw fixation used n(%):  One 3.5-mm 1 (4)  One 4.2-mm 13 (54)  Two 3.5-mm 6 (25)  Two 4.2-mm 2 (8)  One 4.2-mm and one 3.5-mm 1 (4)  Three 3.5-mm 1 (4) | Ankle dorsiflexion, subjective improvement of dorsiflexion  3 months postoperative^a^ |
| Chu 2009^31^ | Before-After Study  USA,  11.2005-05.2007 | N=25  Removal after 8.3 (2-31) months (mean, range) postoperatively | - | Male n (%):  16 (64)  Age [y] mean (range):  11.6 (3-18)  Flexible nail n (%):  Both bone forearm 5 (50) Elbow 3 (30) Wrist 1 (10) Tibia 1 (10)  Plates/Screws n (%): Legg-Calve-Perthes 4 (57) Both bone forearm 1 (14) Development dysplasia of the hip 1 (14) Femur 1 (14)  Screws n (%): Ankle 2 (50) Development dysplasia of the hip 1 (25) Tibia 1 (25)  Staples n (%): Cavus 1 (50) Genu valgum 1 (50)  Screws/rod n (%): Ankle 1 (100)  Staples/Steinmann pin n (%): Cavus 1 (100) | Pain, Pediatric Outcomes Data Collection Instrument  16.5 months postoperative^a^ (mean) |
| Dimitriou 2020^33^ | Before-After Study  Switzerland, Department of Orthopaedics Bürgerspital Solothurn, NR | N=56  Removal of a proximal humeral plate after 7 ± 2 months (mean ± SD) postoperative | - | Male n(%):  17 (30)  Age [y] mean ± SD:  63 ± 12  Affected side n(%):  Right 26 (46)  Dominant 28 (50)  Follow-up (months) mean ± SD  29 (5)  Time to ORIF (Days) mean ± SD  3 (2)  Duration of ORIF (Minutes) mean ± SD  88 (21)  Time to Implant removal (Months) mean ± SD  7 (2)  Duration of Implant removal (Minutes) mean ± SD  39 (8)  Fracture Dislocation n(%):  8 (14%)  Fracture Classification (Neer) n(%):  2-part 10 (18)  3-part 38 (68)  4-part 8 (14) | External rotation, abduction, flexion, subjective increase of function  12 months postoperative^a^  Avascular necrosis, other complications  29 months postoperative^a^ |
| Garner 2015^25^ | Cohort Study  Germany, Trauma Centre  06.2009-06.2014 | N=39  Removal of implants in median at 7.3 months | N=36  Retaining of implants | Male n (%):  22 (53) / 13 (37)  Age [y] mean (range):  49 (44.5-58.5) / 68 (59.5-75.3)  BMI [kg/m²] mean (range):  25.0 (22.6-28.7) / 25.7 (21.4-33.1)  Comorbidities n (%): Diabetes 1 (3) / 1 (3) Hypertension 5 (13) / 7 (19)  Hyperlipidemia 3 (8) / 2 (6)  Peripheral vascular disease 0 / 0  Smoking History 3 (8) / 1 (3)  EtOH abuse 5 (13) / 3 (8)  Time from index procedure until final follow-up [months] mean (range): 15.4 (13.6-26.5) / 40.6 (13.6-57.7)  Schatzker n (%): 1–2 29 (74) / 22 (61) 5–6 10 (26) / 14 (39) | Knee Outcome Survey, lower Extremity Functional Scale, Short Form-36 Survey  15.4/40.6 months (median) |
| Goshima 2019^34^ | Before-After Study  Japan, NR, 01.2012 - 12.2016 | N=101  Removal of implants in mean (SD) 16.4 (5.4) months | - | Male n(%):  32 (32)  Age [y] mean (range):  63.5 (22–83)  BMI [kg/m²] mean (range):  24.2 (16.1–32.3)  Follow-up (months) mean (range):  52.5 (25.4–84.7)  Time to Implant removal (months) mean (range):  16.4 (6.2–36)  Opening width (mm) (range):  11.9 (7.0–19)  Preoperative bearing line ratio (%) (range):  21.5 (− 11.6 to 41.8) | Japanese Orthopedic Association score, Oxford Knee Score, hip–knee–ankle angle, medial proximal tibial angle, posterior tibial slope, weightbearing line ratio  12 months postoperative^a^ |
| Gosling 2005^26^ | Before-After Study  Germany,  01.1987-12.1999 | N=18  Removal of intramedullary nail after 21 months (mean) | - | Male n(%):  48 (70)  Age [y] mean (range):  32 (13-61)  AO-classification n (%): 42-A 19 (30) 42-B 53 (56) 42-C 9 (14) | Complaints improved  7.4 years (mean) |
| Gosling 2004^27^ | Before-After Study  Germany, level I Trauma centre  01.1990-03.1999 | N=51  Removal of intramedullary nail after 27 months (mean) | - |  | Complaints improved  6.3 years (mean) |
| Hamid 2009^28^ | Cohort Study  USA,  2001-2005 | N=18  Removal of implants (e.g. syndesmotic screws) after 13.1 weeks (mean) | N=37  Retaining of implants (e.g. syndesmotic screws) | Male n (%):  8 (53) / 20 (54)  Age [y] mean (range):  47 (21-72)  Weber-classification n (%): Weber-B 3 (20) / 12 (32)  Weber-C 12 (80) / 25 (68) | American Orthopaedic Foot and Ankle Society ankle-hind foot score, pain  30 months postoperative^a^ (mean) |
| Miller 2010^29^ | Before-After Study  USA,  07.2007-01.2008 | N=25  Removal of implants (e.g. syndesmotic screws) after 4.3 months (mean) | - | Male n (%):  11 (44)  Age [y] mean (range): 40 (17-78) | Foot and Ankle Outcome, Olerud-Molander Ankle Score, average range of motion [degree] (mean, p)  7 months postoperative^a^ |
| Tucker 2013^30^ | Cohort Study  UK, level 1 trauma centre practice  01.2008-12.2010 | N=43  Removal of implants (e.g. syndesmotic screws) after 83.44 days (mean) | N=20  Retaining of implants (e.g. syndesmotic screws) | Male n (%):  26 (60.5) / 9 (45)  Age [y] mean (range):  41.84 (16-80) / 45.3 (19-78)  Time of follow-up [months] mean (range):  31.45 (10-43) / 28.57 (26-43)  Time of follow-up [d] mean (range):  83.44 (47–178) / NA | Olerud-Molander Ankle Score, Excellent overall functional outcome grouping  31 months (mean) |
| ^a^= after index surgery  IG=intervention group, CG= control group, SD= standard deviation, NR= not reported, CI= confidence interval, VAS= visual analogue scale, RCT= randomized controlled trial, BMI= body mass index, EtOH= ethanol, NA= not available, ORIF= Open Reduction and Internal Fixation, PAB= pronation abduction, PER= pronation external rotation, SER= supination external rotation | | | | | |
